# Misoprostol administered sublingually at a dose of 12.5 μg versus vaginally at a dose of 25 μg for the induction of full-term labor: a randomized controlled trial protocol

**DOI:** 10.1186/s12978-018-0508-5

**Published:** 2018-04-18

**Authors:** Daniele Sofia Moraes Barros Gattás, José Roberto da Silva Junior, Alex Sandro Rolland Souza, Francisco Edson Feitosa, Melania Maria Ramos de Amorim

**Affiliations:** 1 Instituto de Medicina Integral Prof. Fernando Figueira (IMIP), Recife, Pernambuco Brazil; 2 Department of Maternal and Child Health, Federal University of Pernambuco (UFPE), Recife, Pernambuco Brazil; 3 Assis Chateaubriand Maternity Teaching Hospital, Federal University of Ceará (UFC), Fortaleza, Ceará Brazil; 4 Department of Obstetrics and Gynecology, Federal University of Campina Grande (UFCG), Campina Grande, Paraíba Brazil

**Keywords:** Labor, obstetric, Labor, induced, Misoprostol/administration & dosage administration, sublingual, Multicenter study, Clinical trial

## Abstract

**Background:**

Various methods are currently used for the induction of labor. Nevertheless, the most effective method with the fewest side effects remains to be established. Misoprostol, administered vaginally, has been routinely used for this purpose; however, other forms of administration are being proposed, including the use of sublingual tablets. No studies have yet compared the effectiveness and safety of 12.5-μg misoprostol administered sublingually compared to a 25-μg vaginal dose of the drug for the induction of labor.

**Methods:**

A triple-blind, multicenter, placebo-controlled, randomized clinical trial will be conducted in Brazil at the *Instituto de Medicina Integral Prof. Fernando Figueira* and at the Assis Chateaubriand Maternity Teaching Hospital of the Federal University of Ceará. A total of 140 patients with full-term pregnancies, a live fetus, a Bishop score ≤ 6 and a recommendation of induction of labor will be randomized to one of two groups. One group will receive 12.5-μg sublingual tablets of misoprostol and placebo vaginal tablets, while the other group will receive placebo sublingual tablets and vaginal tablets containing 25 μg of misoprostol. The principal endpoint is the rate of tachysystole. The secondary endpoints are vaginal delivery within 24 h of induction, uterine hyperstimulation, Cesarean section, severe neonatal morbidity or perinatal death, severe maternal morbidity or maternal death, and maternal preference regarding the route of administration of the drug. Student’s t-test, and the chi-square test of association or Fisher’s exact test, as appropriate, will be used in the data analysis. Risk ratios and their respective 95% confidence intervals will be calculated.

**Discussion:**

Misoprostol has been identified as a safe, inexpensive, easily administered option for the induction of labor, with satisfactory results. An experimental study has shown that misoprostol administered sublingually at a dose of 25 μg appears to be effective and is associated with greater maternal satisfaction when labor is induced in women with an unfavorable cervix. Nevertheless, the rate of tachysystole remains high; therefore, further studies are required to determine the ideal dose and the ideal interval of time between doses.

**Trial registration:**

ClinicalTrial.gov, NCT01406392.

## Plain summary

Various methods are currently used for the induction of labor. Nevertheless, the most effective method with the fewest side effects remains to be established. Misoprostol, administered vaginally, has been routinely used for this purpose; however, other forms of administration are being proposed, including the use of sublingual tablets. No studies have yet compared the effectiveness and safety of 12.5-μg misoprostol administered sublingually compared to a 25-μg vaginal dose of the drug for the induction of labor. A triple-blind, multicenter, placebo-controlled, randomized clinical trial will be conducted in Brazil at the *Instituto de Medicina Integral Prof. Fernando Figueira* and at the Assis Chateaubriand Maternity Teaching Hospital of the Federal University of Ceará. A total of 140 patients with full-term pregnancies, a live fetus, a Bishop score ≤ 6 and a recommendation of induction of labor will be randomized to one of two groups. One group will receive 12.5-μg sublingual tablets of misoprostol and placebo vaginal tablets, while the other group will receive placebo sublingual tablets and vaginal tablets containing 25 μg of misoprostol. The principal endpoint is the rate of tachysystole.

## Background

Induction of labor refers to any procedure used to stimulate uterine contraction before it occurs spontaneously [1]. When the continuation of pregnancy represents a risk to the mother and/or the fetus that is greater than the risk of interrupting the pregnancy, labor induction is an option to allow vaginal delivery to occur [2].

Misoprostol is a synthetic analog of prostaglandin E1 that acts on the cervix and on the uterine smooth muscle, facilitating cervical dilatation and promoting uterine contractions [3]. The first time misoprostol was used for labor induction involving a live fetus was in 1991, and the drug was administered vaginally [4]. Since then, various studies have been conducted using different dosage regimens and progressively lower doses. In addition, some studies have compared misoprostol with other methods of labor induction, while others have compared different routes of administration [5–8].

Currently, the most commonly used and recommended routes of administration of misoprostol for labor induction in routine practice are the vaginal and oral routes at a dose of 25 μg [6, 7, 9]; however, the sublingual and buccal routes have also been proposed [5, 8].

The administration of misoprostol by the sublingual route was proposed as a means of reducing the number of vaginal examinations, thus providing patients with greater comfort and possibly also reducing rates of maternal and fetal infection [10]. In addition, evaluation of the pharmacokinetics of the drug showed a higher peak of plasma concentration in pregnant women receiving misoprostol by the sublingual route compared to oral or vaginal administration or to vaginal administration with the addition of water. The bioavailability of the drug is also greater with the use of the sublingual route of administration [3]. On the other hand, plasma levels are maintained over a longer period of time when the vaginal route is used [3].

In a systematic review that included five clinical trials comparing misoprostol administered sublingually and vaginally, no statistically significant difference was found between the two groups in relation to the rate of vaginal deliveries within the first 24 h, to the rate of uterine hyperstimulation or to the Cesarean section rate. There was, however, a greater risk of tachysystole in the sublingual misoprostol group [10]. Those authors also suggested that this effect was dose-dependent; i.e. the higher the dose of misoprostol, the greater the risk of tachysystole. In that study, the lowest dose used was 25 μg [10]. Therefore, those authors concluded that new studies would have to be conducted to determine the lowest effective dose with the fewest side effects [10].

This study was proposed with the objective of comparing the effectiveness and safety of the sublingual administration of 12.5 μg of misoprostol (low dose) with the vaginal administration of 25 μg of misoprostol (standard recommended dose) for the induction of labor involving a live, full-term fetus.

## Methods/design

### Setting and recruitment

The study will be developed at the *Instituto de Medicina Integral Prof. Fernando Figueira* and at the Assis Chateaubriand Maternity Teaching Hospital of the Federal University of Ceará, all of which are situated in northeastern Brazil. These institutions provide integrated women’s healthcare that encompasses primary, secondary and tertiary healthcare. They operate as university teaching hospitals, receiving students from the Medical and Nursing Schools, and also have a medical residency program in Obstetrics and Gynecology and postgraduate programs. In each institute, around 400–450 births take place every month, with induction of labor being indicated in approximately 10–20% of cases.

### Participant selection

The attending obstetrician will identify possible candidates for the study. An ultrasound scan will be performed and a checklist will be completed to evaluate whether the candidate fulfills the eligibility criteria for inclusion in the study. If the woman is eligible, she will then be provided with explanations regarding the reason for conducting the study and its importance.

All the patients included in the study will be provided with information regarding the objectives and possible consequences of their participation. If they voluntarily agree to participate, they will then be asked to sign an informed consent form and will be referred for antepartum care under the responsibility of the investigators and the duty obstetricians.

### Study design

A multicenter, triple-blind, randomized, controlled clinical trial will be conducted.

### Inclusion criteria

Gestational age ≥ 37 weeks; live fetus; cephalic vertex presentation; Bishop score ≤ 6; and the presence of clinically satisfactory fetal vitality.

### Exclusion criteria

Ultrasound-estimated fetal weight ≥ 4000 g; amniotic fluid index < 5; previous Cesarean section; previous uterine scar from a myomectomy or other uterine surgery; genital bleeding of unexplained origin; fetal anomalies; tumors, malformations and/or ulcerations in the vulvoperineal region and birth canal that could be harmful to the mother and/or fetus during labor; and chorioamnionitis.

### Sample size

Predicting a frequency of tachysystole of 6,7% % in the 12.5-μg sublingual misoprostol group (pilot study) and of 13.2% in the 25-μg vaginal misoprostol group [7], for a 95% confidence interval and a power of 80%, 70 women would have to be recruited to each group.

### Primary endpoint

Tachysystole.

### Secondary endpoints

Vaginal delivery within 24 h of induction; uterine hyperstimulation; Cesarean section; maternal preference regarding the route of administration of the drug; severe neonatal morbidity (convulsions and neonatal asphyxia) or perinatal death; severe maternal morbidity (uterine rupture, sepsis or admission to the intensive care unit) or maternal death; need for oxytocin; number of doses of misoprostol required to trigger labor; time between the first dose and the beginning of labor and delivery; failed induction; need for epidural anesthesia; operative vaginal delivery; maternal side effects (nausea, vomiting, diarrhea, postpartum hemorrhage and fever); meconium in the amniotic fluid; non-reassuring fetal heart rate; 1st and 5th minute Apgar scores < 7; admission of the newborn infant to the neonatal intensive care unit; and need for neonatal resuscitation.

### Randomization and preparation of the medications

The Random Allocation software program, version 1.0.0 (Isfahan, Iran) will be used to randomize participants in the study to labor induction with sublingual or vaginal misoprostol, using the letters A and B, with no prior knowledge or indication of what each letter means. The meaning of the letters will be defined in a random draw conducted by the pharmacist. Neither the investigators nor the statistician will have access to that information. The pharmacist responsible for preparing the medication will place the sublingual misoprostol tablets + the placebo vaginal tablets or the placebo sublingual tablets + the misoprostol vaginal tablets in standardized boxes in accordance with the randomization list. Everyone else in the study (investigators, statistician and participants) will remain blinded to this information until data analysis is complete.

Identical boxes will be prepared and numbered sequentially from 1 to 140 in accordance with the randomization table. Each box will contain a set of eight vaginal tablets (placebo or 25 μg of misoprostol) and eight sublingual tablets (placebo or 12.5 μg of misoprostol), thus guaranteeing the triple-blind design of the study, since neither the investigators nor the pregnant women nor the statistician will be aware of the contents of the boxes.

The vaginal and sublingual placebo and misoprostol tablets will be produced by the pharmaceutical company *Hebron Indústrias Químicas e Farmacêuticas*. The vaginal tablets of misoprostol consist of misoprostol, together with lactose, microcrystalline cellulose, aerosil, explocel, sorbitol and talc, and are sold commercially under the brand name Prostokos^®^ (25 μg). For the purposes of the present study, the aforementioned pharmaceutical company will prepare 12.5-μg tablets of misoprostol for sublingual use. The placebo vaginal and sublingual tablets will be identical in shape, size, color, smell, taste and weight to those containing the active drug prepared specifically for the study, and will be stored in indistinguishable cardboard boxes.

### Drug administration

Induction of labor will be programmed to begin in the morning, preferably at 6 am, with treatment being interrupted overnight at 10 pm if labor does not begin in the first 12 h. Induction will be considered to have failed if labor has not begun six hours after the eighth and final dose of misoprostol.

The attending physician will place a sublingual tablet of 12.5 μg of misoprostol or a placebo tablet under the woman’s tongue every six hours. The attending physician will insert a vaginal tablet of 25 μg of misoprostol or a placebo tablet into the woman’s posterior uterine fundus every six hours. The patients will be aware of the time at which they will be given the medication.

**Fig. 1:**
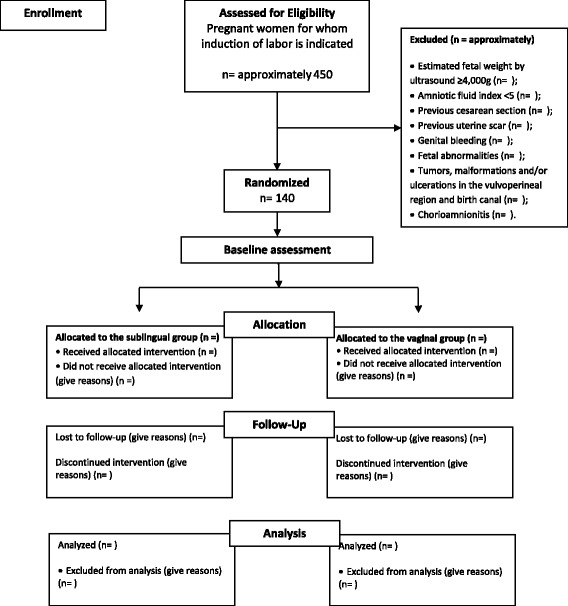
Flowchart of procedures for participant selection and monitoring (CONSORT) [12]

A summary of the study design is shown in Fig. 1.

### Analysis

The statistical analysis will be conducted using the Epi Info statistical software program, version 7 (Centers for Disease Control and Prevention, Atlanta, USA), with the groups being identified only as A and B. The identification of the groups will only be revealed once the results have been obtained and tables have been prepared. The categorical variables will be compared in contingency tables using the chi-square test of association and Fisher’s exact test, as pertinent. Risk ratios will be calculated as measures of relative risk, together with their respective 95% confidence intervals. The number needed to treat (NNT) to measure the clinical benefit or harm of the treatments will be calculated, together with their respective 95% confidence intervals.

In relation to the continuous quantitative variables with dissimilar variances, if distribution is normal, the groups will be compared using Student’s t-test for non-paired samples (parametric tests). If distribution is found not to be normal, the Mann-Whitney non-parametric test will be used. These tests will be used to identify specific differences between the two groups.

## Discussion

The technological progress achieved in modern obstetrics permits effective techniques to be used to diagnose maternal and fetal situations requiring early elective delivery. One of the principal limiting factors to this process is the condition of unfavorable cervix, which hampers induction of labor, consequently contributing towards increasing Cesarean section rates.

Misoprostol has been identified as a safe, inexpensive, easily administered option for the induction of labor, with satisfactory results. An experimental study showed that sublingual misoprostol at a dose of 25 μg appears to be effective and is associated with greater maternal satisfaction when used to induce labor in women with an unfavorable cervix. Nevertheless, the rate of tachysystole remains high; therefore, further studies need to be conducted to determine the dose and the ideal time interval between doses to minimize the side effects. The only clinical trial conducted using a 12.5-μg dose of misoprostol administered sublingually, a non-randomized open study with no control group, reported that labor was successfully triggered in 90% of cases, with vaginal deliveries occurring in 60% of the women, and a rate of 47% of vaginal deliveries within 24 h [11]. When the frequency of tachysystole found in that study is compared with others in which misoprostol was used vaginally at a dose of 25 μg, a similar frequency of between 4.3 and 6.6% is found [10]. The systematic review available in the Cochrane Library suggests a global rate of uterine hyperstimulation with no changes in fetal heart rate of 11% with 25 μg of misoprostol, even higher than that found in an earlier review [7]. Nevertheless, these results are based on a single clinical trial with no control group, thus rendering comparisons difficult.

We believe that with this low dose of misoprostol administered sublingually the frequency of tachysystole or uterine hyperstimulation may be lower than that found with the habitual 25 μg dose of misoprostol administered vaginally, since it was suggested in a systematic review that the occurrence of tachysystole with misoprostol is probably dose-dependent [11].

This will be the first randomized, controlled clinical trial to compare the effectiveness and safety of misoprostol at a dose of 12.5 μg administered sublingually with the currently standard 25 μg dose administered vaginally every six hours to induce labor in pregnant women with a full-term live fetus. Expectations are that the present study will clarify whether the rate of tachysystole during labor induction is lower in the sublingual group. On the other hand, no differences between the groups are expected with respect to uterine rupture, need for analgesia during labor, need for operative vaginal delivery or the rate of maternal death.

### Trial status

This trial is registered at ClinicalTrials.gov as: NCT01406392. Recruitment commenced in August 2015 and is expected to continue until July 2018.
